# Supplementary material for: Structural mechanisms of assembly, gating, and calmodulin modulation of human olfactory CNG channel
Source: Nat Commun. 2025 Oct 23;16:9380. doi: 10.1038/s41467-025-64436-5 (PMC12549891; doi:10.1038/s41467-025-64436-5)
Supplement: Supplementary file 1 — Supplementary Information [file 41467_2025_64436_MOESM1_ESM.pdf]

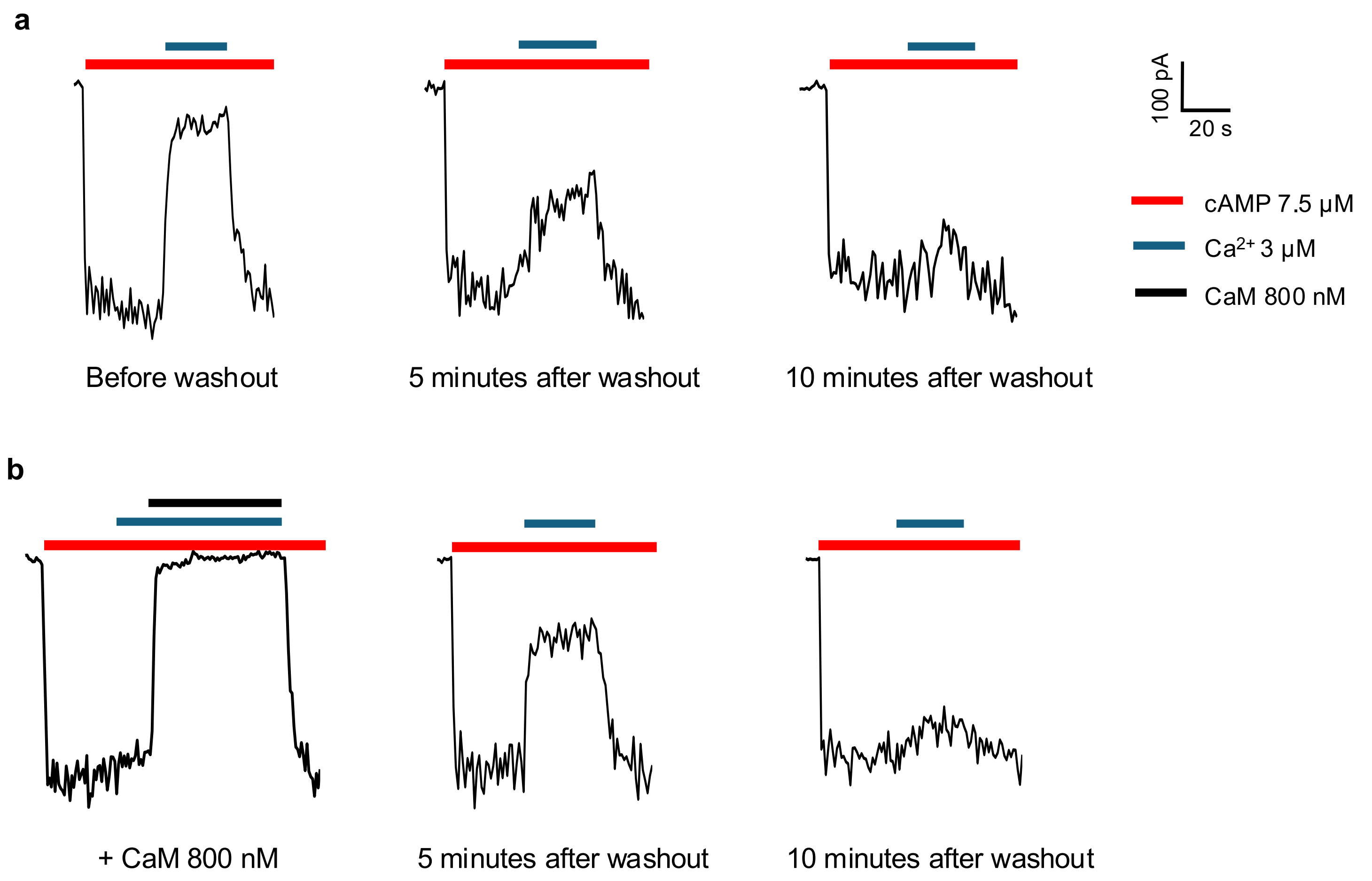

Supplementary Fig. 1 High-affinity binding of CaM to CNGA2/A4/B1b complex.

a, Sample traces of endogenous CaM-mediated cytosolic Ca^{2+} inhibition of CNGA2/A4/B1b recorded in excised patch. Inward current at -100 mV was elicited by 7.5 µM cAMP. Shown are traces recorded before and after washout with EGTA-containing bath solution, indicating that the removal of bound endogenous CaM requires a long washout without Ca^{2+}.

b, Sample traces of Ca^{2+}/CaM inhibition of CNGA2/A4/B1b recorded using the same patch as **(a)** after 10 min washout with EGTA to remove the endogenous CaM. The first trace demonstrates that the channel can regain its Ca^{2+}-induced inhibition by supplementing purified CaM protein. The second and third traces show the Ca^{2+}-induced inhibition after 5- and 10-minute washout, respectively, with EGTA-containing bath solution, demonstrating that the supplemented CaM also binds to the channel with high affinity, and requires a long washout to be removed.

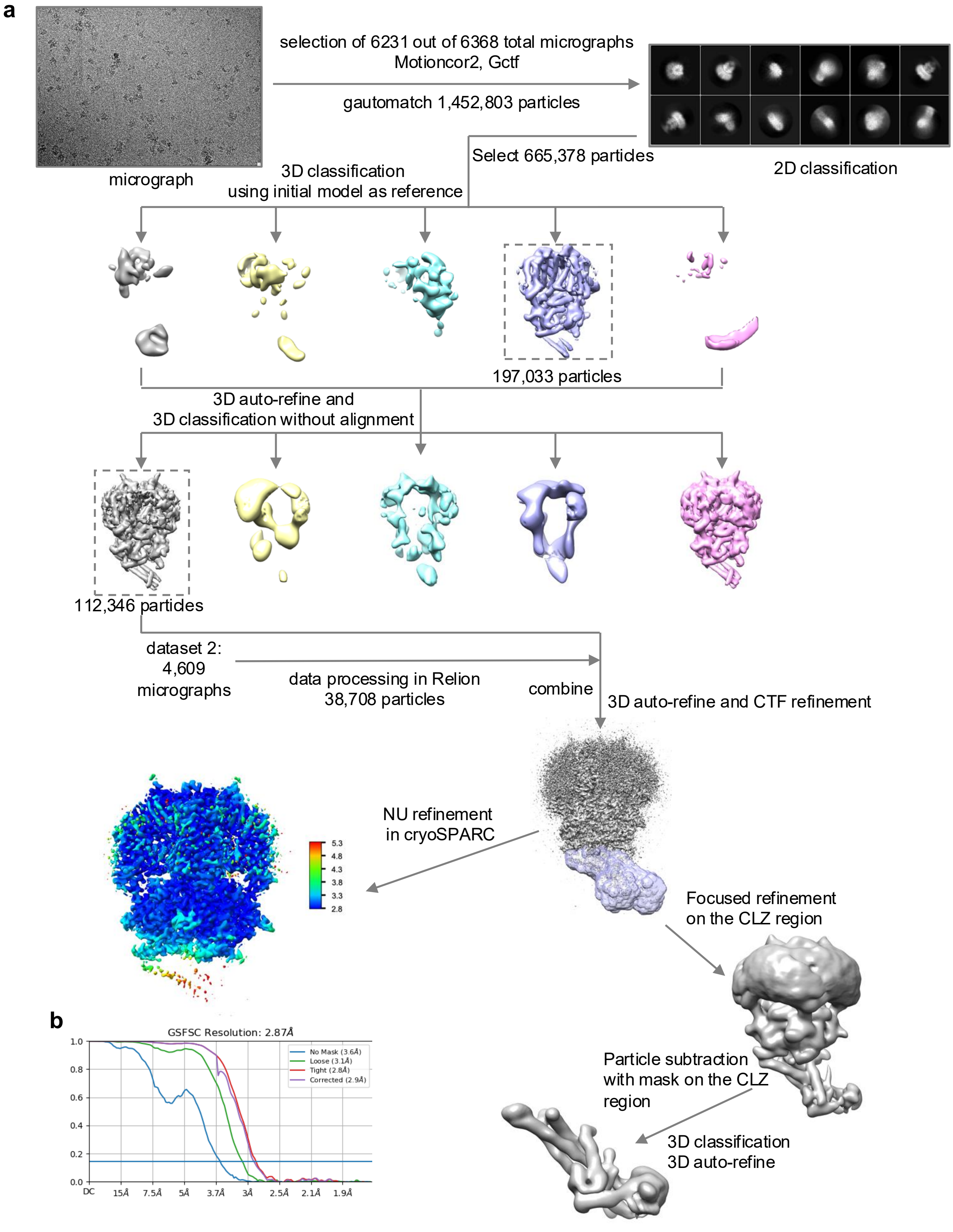

Supplementary Fig. 2 Cryo-EM data processing scheme of CNGA2/A4/B1b-CaM complex.
a, Representative micrograph and flow chart of the cryo-EM data processing procedure. Selected 2D class averages are shown.
b, Fourier Shell Correlation curves showing the overall resolution at FSC=0.143.

a

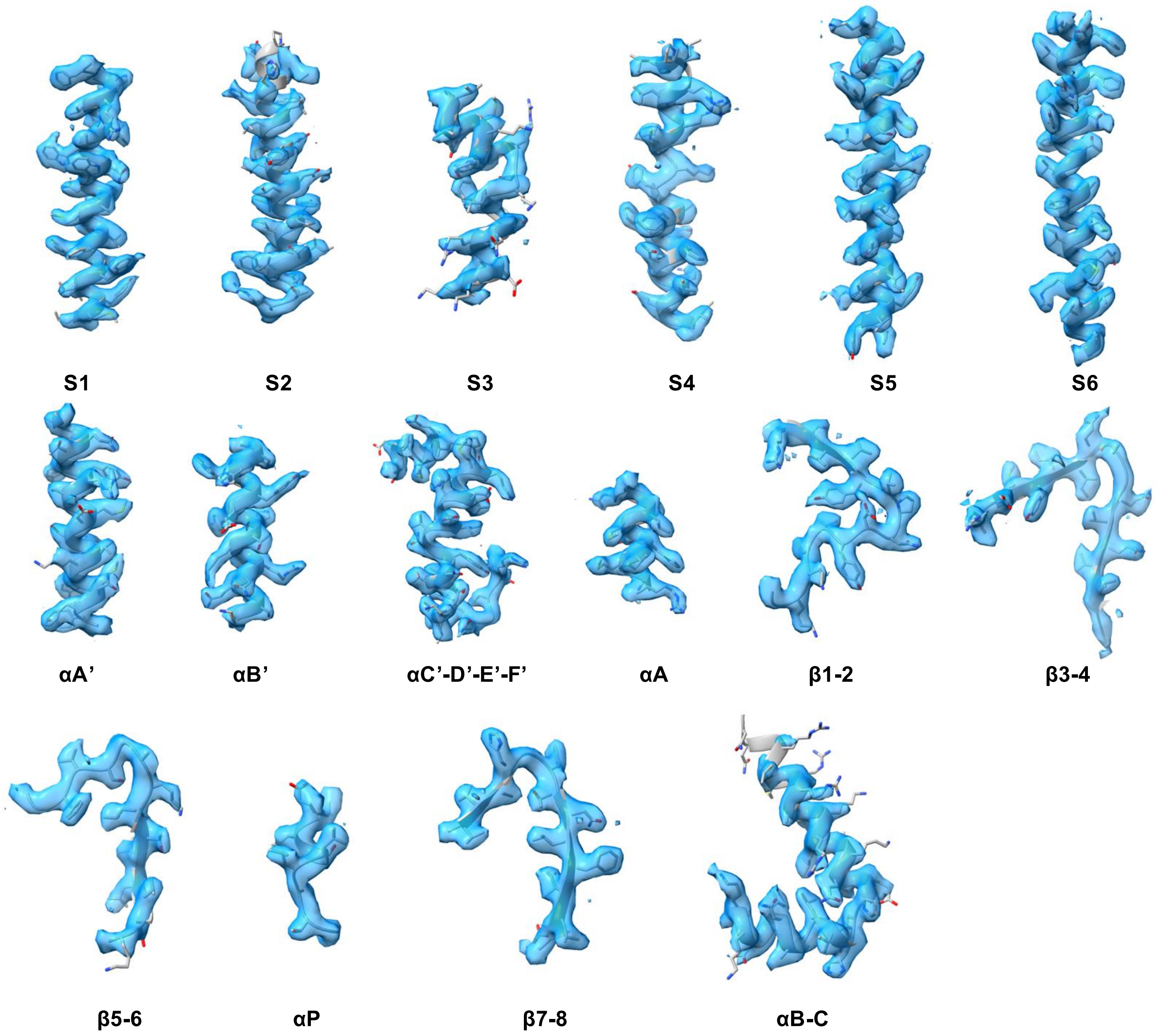

b

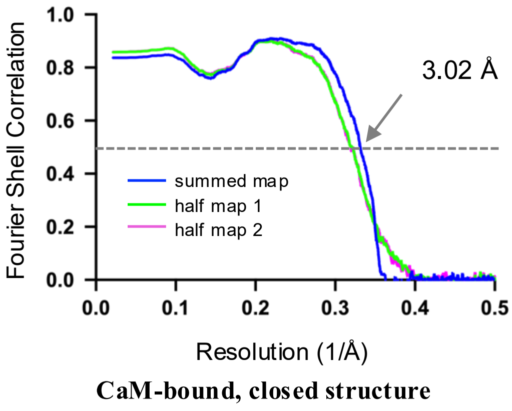

Supplementary Fig. 3 Sample density maps of the human CNGA2/A4/B1b-CaM complex.

a, Density maps of the CNGB1b subunit contoured at the threshold level of 0.52 using the ChimeraX software.

b, The Fourier shell correlation (FSC) curves for cross-validation between the maps and the model: model versus the summed map in blue (sum), model versus the half map in green, model versus the half map not used for refinement in pink.

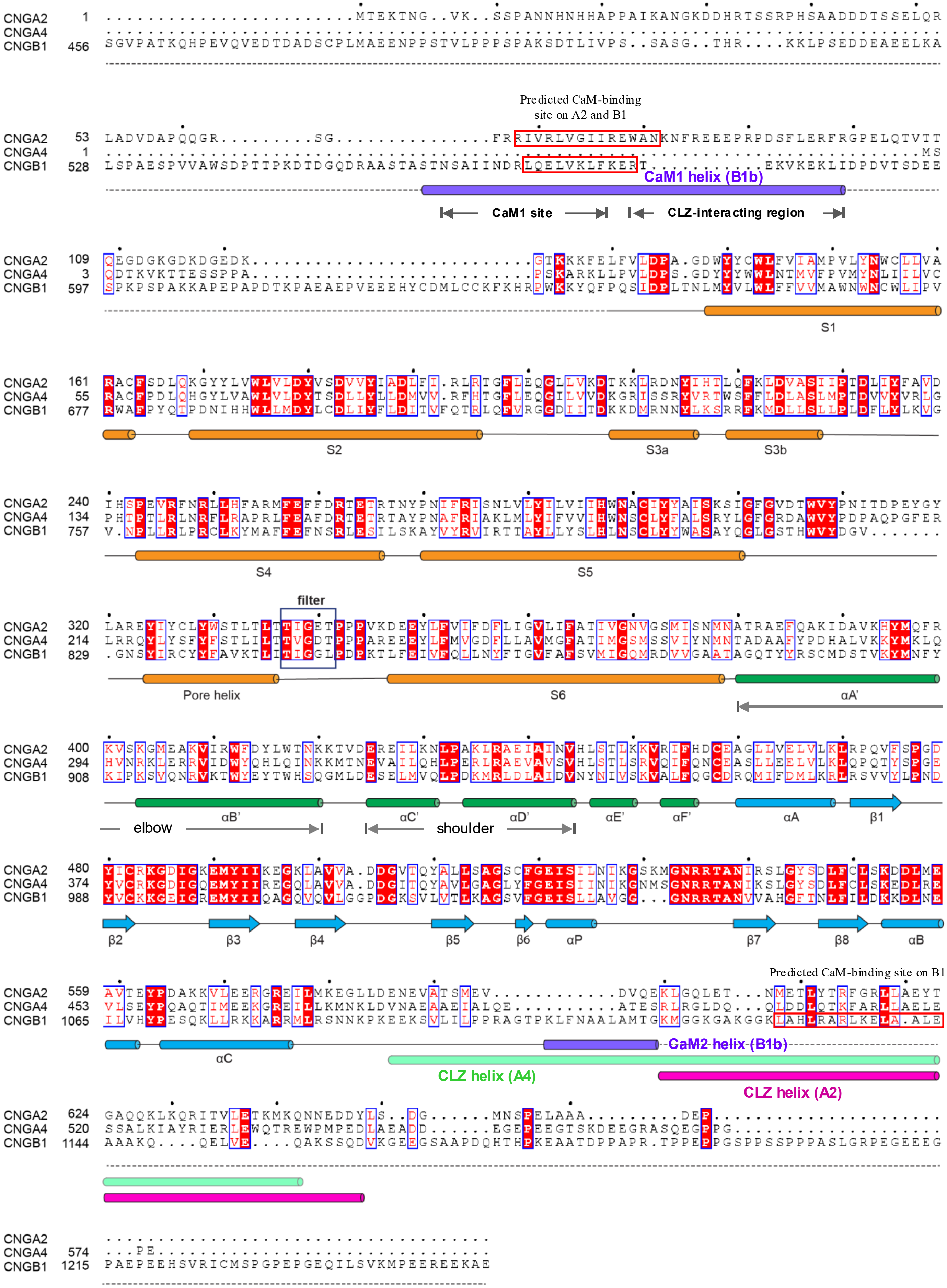

Supplementary Fig. 4 Sequence alignment of human CNGA2, A4, and B1b.

Secondary structure assignments are based on the structure of CNGA2/A4/B1b-CaM complex. Red boxes enclosed the predicted sequences of CaM-binding sites in CNGA2 and CNGB1b.

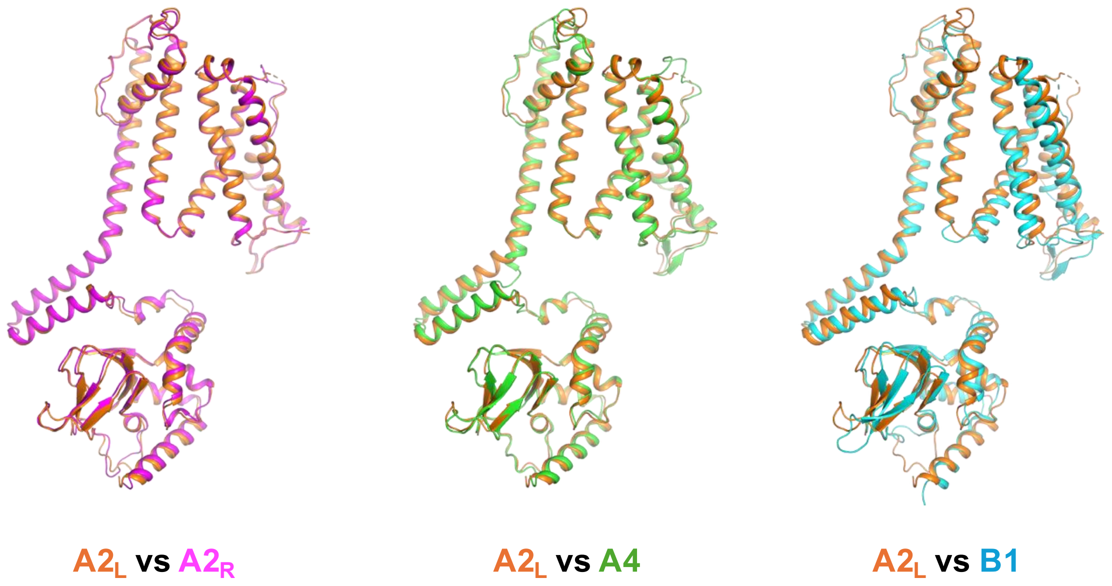

Supplementary Fig. 5. Structural comparison between A2$_L$ and other CNG subunits from the CNGA2/A4/B1b-CaM complex.

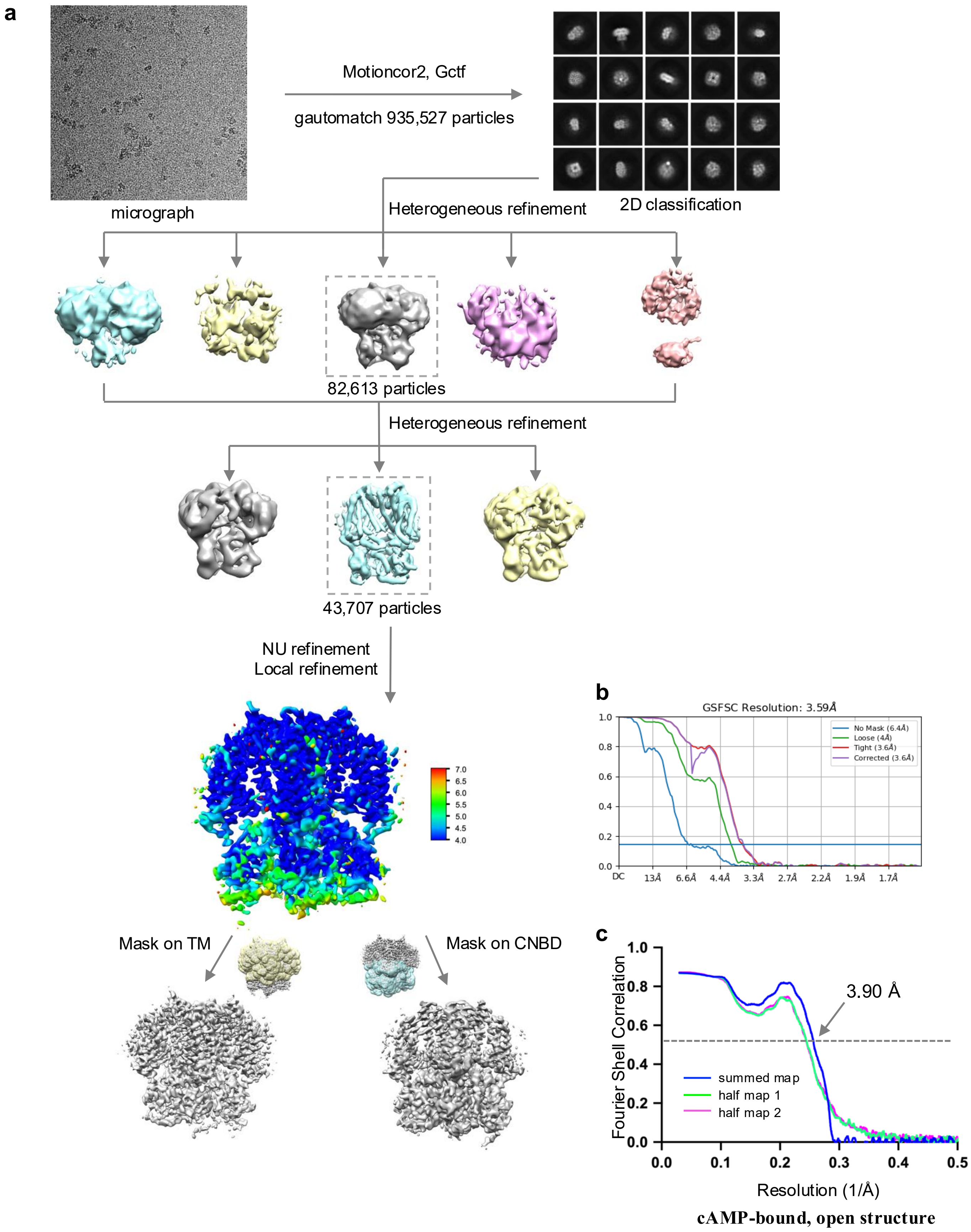

Supplementary Fig. 6 Cryo-EM data processing scheme of cAMP-bound CNGA2/A4/B1b.

a, Representative micrograph and flow chart of the cryo-EM data processing procedure. Selected 2D class averages are shown.

b, Fourier Shell Correlation curves showing the overall resolution at FSC=0.143.

c, The Fourier shell correlation (FSC) curves for cross-validation between the maps and the model: model versus the summed map in blue (sum), model versus the half map in green, model versus the half map not used for refinement in pink.

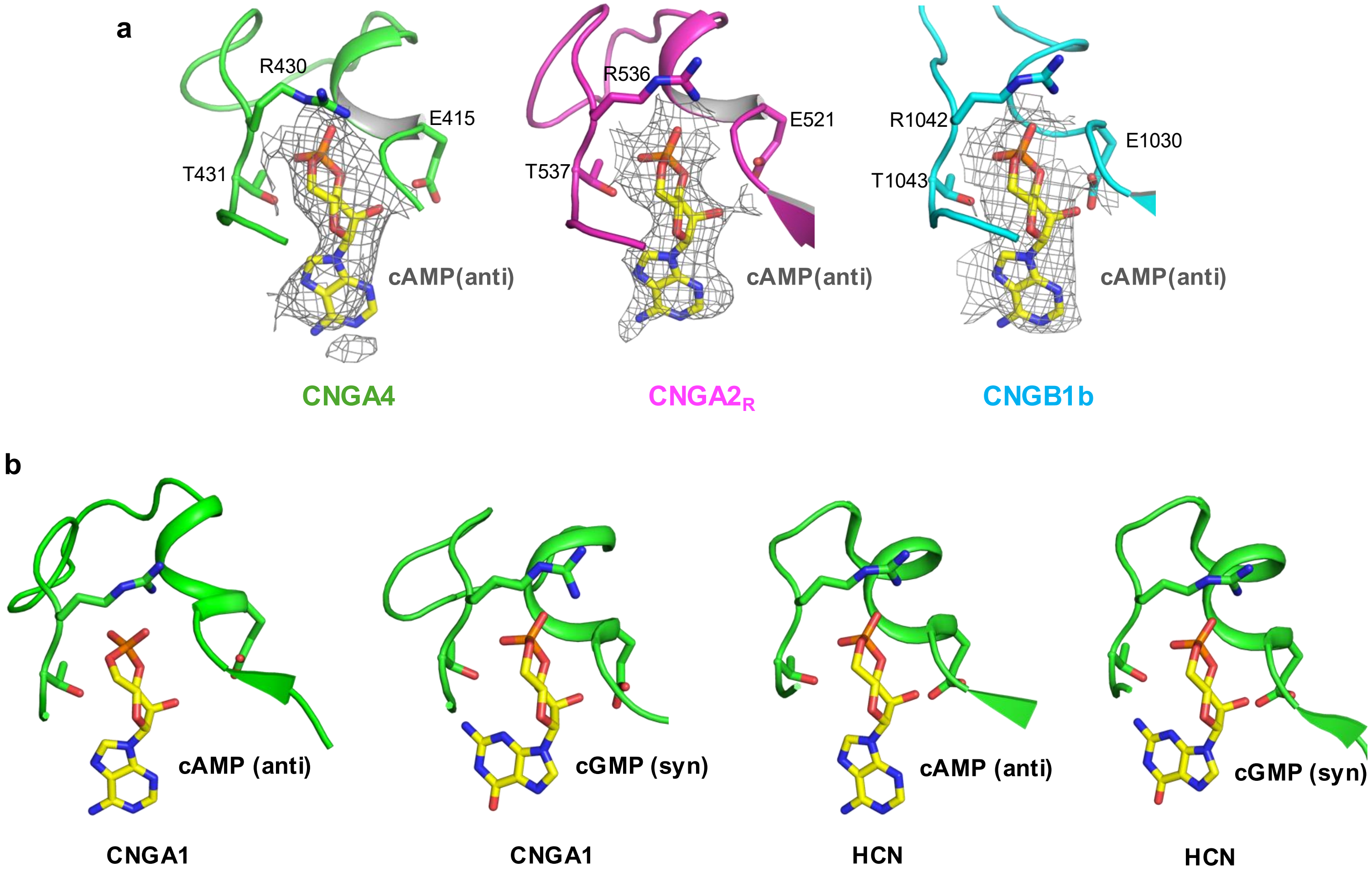

Supplementary Fig. 7 cNMP binding in CNG and HCN channels.

a, cAMP binding in CNGA4, A2$_R$, and B1b subunits. The density maps for cAMP (in anti conformation) are contoured at 6σ.

b, cAMP and cGMP binding in CNGA1 and HCN channels.

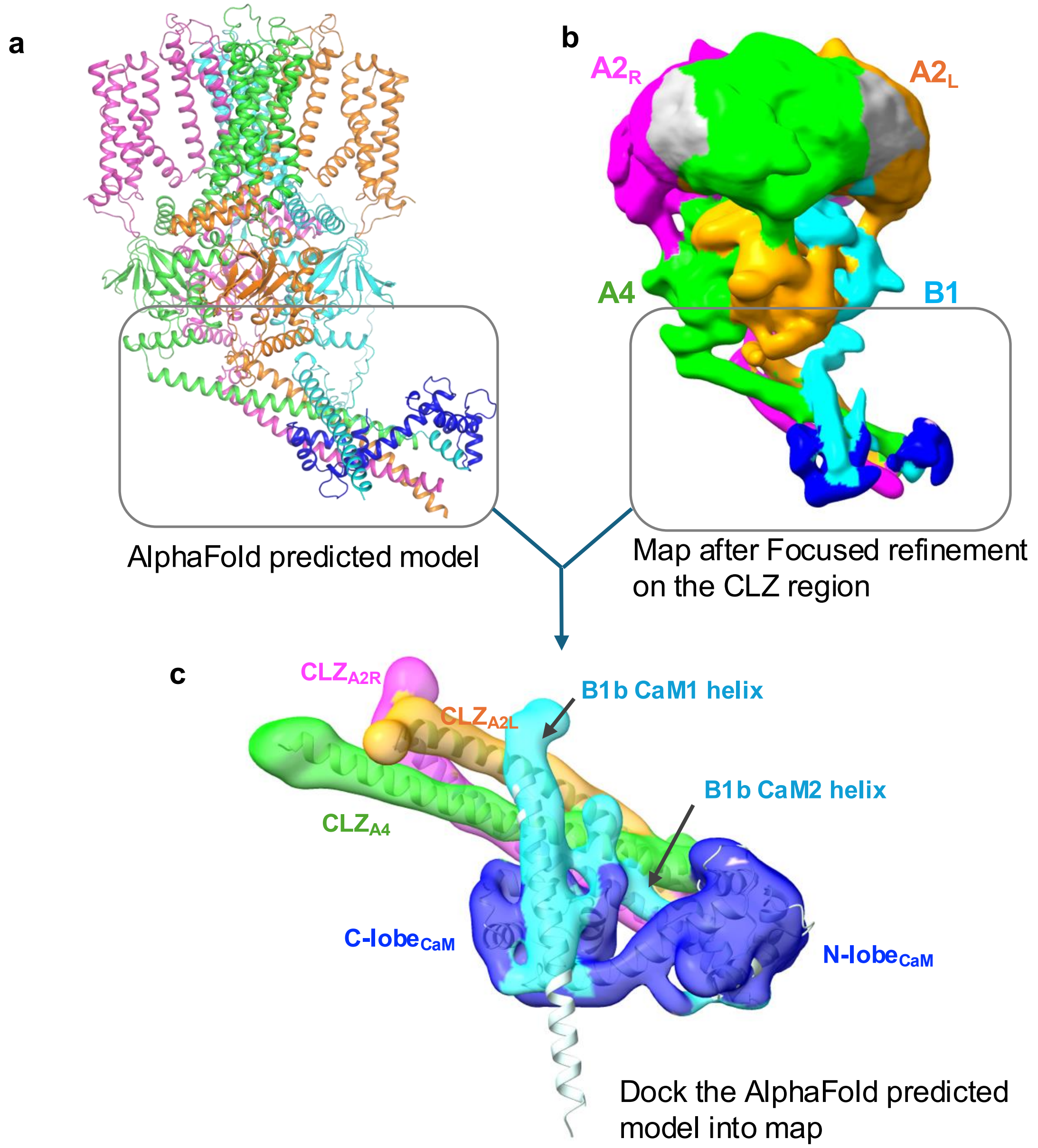

d

Rank	Id	Protein1	Protein2	AbsPos1	AbsPos2	ld-Score	FDR
1	LKELAALEAAAKQQELVEQAK-VFDKDGNGYISAAELR-a12-b4	sp|Q14028|CNGB1	sp|P0DP25|CALM3	1147	95	54.58	0
2	GLDQQLDDLQTKFAR-KVLEER-a12-b1	sp|Q8IV77|CNGA4	sp|Q16280|CNGA2	510	568	50.56	0
3	EAFSLFDKDGDGTITTK-LKELAALEAAAK-a8-b2	sp|P0DP25|CALM3	sp|Q14028|CNGB1	22	1137	46.18	0
4	VFDKDGNGYISAAELR-GGKLAHLR-a4-b3	sp|P0DP25|CALM3	sp|Q14028|CNGB1	95	1128	44.64	0
5	LKELAALEAAAK-KVLEER-a2-b1	sp|Q14028|CNGB1	sp|Q16280|CNGA2	1137	568	44.45	0
6	VFDKDGNGYISAAELR-KKLPSEDDEAEELK-a4-b2	sp|P0DP25|CALM3	sp|Q14028|CNGB1	95	514	43.86	0
7	ELAALEAAAKQQELVEQAK-VFDKDGNGYISAAELR-a10-b4	sp|Q14028|CNGB1	sp|P0DP25|CALM3	1147	95	42.38	0
8	GLDQQLDDLQTKFAR-LKELAALEAAAK-a12-b2	sp|Q8IV77|CNGA4	sp|Q14028|CNGB1	510	1137	41.47	0
9	LKELAALEAAAK-KGDIGK-a2-b1	sp|Q14028|CNGB1	sp|Q16280|CNGA2	1137	484	41.09	0
10	TGFLEQGLLVKDTK-LKELAALEAAAK-a11-b2	sp|Q16280|CNGA2	sp|Q14028|CNGB1	206	1137	40.88	0
11	TANVVAHGFTNLFILDKK-KVLEER-a17-b1	sp|Q14028|CNGB1	sp|Q16280|CNGA2	1059	568	40.42	0
12	EAFSLFDKDGDGTITTK-GAKGGK-a8-b3	sp|P0DP25|CALM3	sp|Q14028|CNGB1	22	1125	39.33	0
13	VFDKDGNGYISAAELR-LKELAALEAAAK-a4-b2	sp|P0DP25|CALM3	sp|Q14028|CNGB1	95	1137	39.25	0
14	EAFSLFDKDGDGTITTK-LFNAALAMTGKMGGK-a8-b11	sp|P0DP25|CALM3	sp|Q14028|CNGB1	22	1118	39.16	0

Supplementary Fig. 8 Modeling the assembly between CaM and the C-terminal CLZ region of CNGA2/A4/B1b.

a, AlphaFold-predicted structure of CNGA2/A4/B1b-CaM complex. The unstructured regions are removed for clarity.

b, EM map after focused 3D reconstruction of CNGA2/A4/B1b-CaM complex with mask around the C-terminal region (boxed region).

c, Docking of the AlphaFold-predicted structure into the EM-map at the CLZ region after focused refinement.

d, List of the top inter-subunit cross-linking sites identified from the cross-linking mass spectrometry analysis of the CNGA2/A4/B1b-CaM complex. The cross-linking reactions between CaM and CNGB1b are highlighted in green.

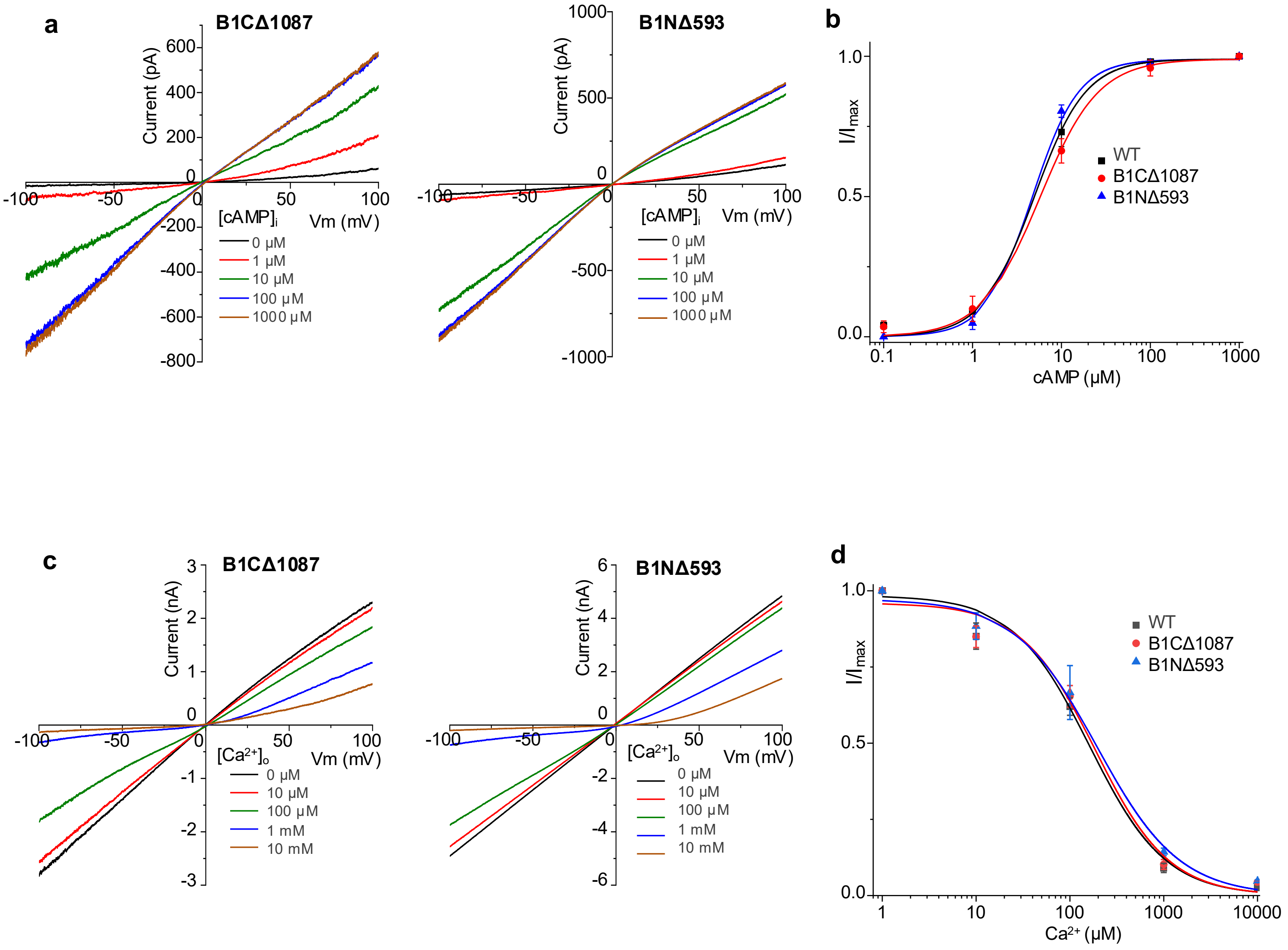

Supplementary Fig. 9 Confirmation of 2:1:1 subunit assembly of the two loss-of-Ca^{2+}/CaM inhibition olfactory CNG mutants with N- or C-terminal deletion in the CNGB1b subunit.

a, Sample I-V curves of B1CΔ1087 and B1NΔ593 mutants recorded in excised patches with varying cAMP concentrations in the bath (cytosolic).

b, Comparison of the concentration-dependent cAMP activation at 100 mV between WT and mutant channels. The activation curves are least square fits to the Hill equation with EC_{50} = 4.9 ± 0.5 μM and n = 1.51 ± 0.15 for WT, EC_{50} = 5.7 ± 0.4 μM and n = 1.32 ± 0.11 for B1CΔ1087, and EC_{50} = 4.6 ± 0.2 μM and n = 1.62 ± 0.11 for B1NΔ593. Data points are mean ± SEM (n = 5 independent replicates).

c, Sample I-V curves of B1CΔ1087 and B1NΔ593 mutants recorded using patch clamp in whole-cell configuration with varying Ca^{2+} concentrations in the bath (extracellular). The pipette solution contains 1 mM cAMP.

d, Comparison of the concentration-dependent Ca^{2+} inhibition at -100 mV between WT and mutant channels. The inhibition curves are least square fits to the Hill equation with K_i = 161.7 ± 40.9 μM and n = 1.56 ± 0.36 for WT, K_i = 185.8 ± 54.8 μM and n = 1.11 ± 0.31 for B1CΔ1087, and K_i = 195.9 ± 43.1 μM and n = 0.99 ± 0.17 for B1NΔ593. Data points are mean ± SEM (n = 5 independent replicates). Source data are provided as a Source Data file.

	CaM-bound, closed state<br>EMDB: EMD-64395<br>PDB: 9UPG	cAMP-bound, open state<br>EMDB: EMD-64394<br>PDB: 9UPF
**Data collection and processing**		
Magnification	105,000	165,000
Voltage (kV)	300	300
Electron exposure ($e^{-}/Å^{2}$)	60	60
Defocus range (μm)	-0.8 ~ -1.8	-0.8 ~ -1.8
Pixel size (Å)	0.83	0.738
Symmetry imposed	C1	C1
Initial particle images (no.)	2,152,711	935,527
Final particle images (no.)	151,054	43,707
Map resolution (Å)<br>FSC threshold	2.87<br>0.143	3.59<br>0.143
**Refinement**		
Initial model used<br>(PDB code)	7RH9	7RHI
Model resolution (Å)<br>FSC threshold	3.02<br>0.5	3.90<br>0.5
Model composition<br>Non-hydrogen atoms<br>Protein residues<br>Ligands	 17912<br>2202<br>6: Ca	 14581<br>1774<br>2: Ca<br>4: CMP
B factors ($Å^{2}$)<br>Protein<br>Ligands	 22.99<br>57.21	 65.56<br>83.11
R.m.s. deviations<br>Bond lengths (Å)<br>Bond angles (°)	 0.005<br>0.629	 0.005<br>0.847
Validation<br>MolProbity score<br>Clashscore<br>Poor rotamers (%)	 1.21<br>4.26<br>0	 1.25<br>4.75<br>0.06
Ramachandran plot<br>Favored (%)<br>Allowed (%)<br>Disallowed (%)	 99.04<br>0.96<br>0	 98.92<br>1.08<br>0

Supplementary Table 1. Cryo-EM data collection and model statistics.
