# Supplementary material for: Structural mechanisms of assembly, gating, and calmodulin modulation of human olfactory CNG channel
Source: Nat Commun. 2025 Oct 23;16:9380. doi: 10.1038/s41467-025-64436-5 (PMC12549891; doi:10.1038/s41467-025-64436-5)
Supplement: Supplementary file 2 — Description of Additional Supplementary Files [file 41467_2025_64436_MOESM2_ESM.pdf]

Description of Additional Supplementary Files

File name: Supplementary Movie 1
Description: Inter-subunit relay of cAMP-induced conformational changes. The movie consists of four segments, each depicting how cAMP binding drives conformational relays between neighboring subunits in the CNGA2/A4/B1b channel.
