# Supplementary material for: Structural mechanisms of assembly, gating, and calmodulin modulation of human olfactory CNG channel
Source: Nat Commun. 2025 Oct 23;16:9380. doi: 10.1038/s41467-025-64436-5 (PMC12549891; doi:10.1038/s41467-025-64436-5)
Supplement: Supplementary file 4 — Reporting Summary [file 41467_2025_64436_MOESM4_ESM.pdf]

nature portfolio

Corresponding author(s): Youxing Jiang

Last updated by author(s): Aug 15, 2025

Reporting Summary

Nature Portfolio wishes to improve the reproducibility of the work that we publish. This form provides structure for consistency and transparency in reporting. For further information on Nature Portfolio policies, see our Editorial Policies and the Editorial Policy Checklist.

Statistics

For all statistical analyses, confirm that the following items are present in the figure legend, table legend, main text, or Methods section.

n/a	Confirmed	
☐	☒	The exact sample size (*n*) for each experimental group/condition, given as a discrete number and unit of measurement
☐	☒	A statement on whether measurements were taken from distinct samples or whether the same sample was measured repeatedly
☐	☒	The statistical test(s) used AND whether they are one- or two-sided<br>*Only common tests should be described solely by name; describe more complex techniques in the Methods section.*
☒	☐	A description of all covariates tested
☒	☐	A description of any assumptions or corrections, such as tests of normality and adjustment for multiple comparisons
☐	☒	A full description of the statistical parameters including central tendency (e.g. means) or other basic estimates (e.g. regression coefficient) AND variation (e.g. standard deviation) or associated estimates of uncertainty (e.g. confidence intervals)
☐	☒	For null hypothesis testing, the test statistic (e.g. *F*, *t*, *r*) with confidence intervals, effect sizes, degrees of freedom and *P* value noted<br>*Give P values as exact values whenever suitable.*
☒	☐	For Bayesian analysis, information on the choice of priors and Markov chain Monte Carlo settings
☒	☐	For hierarchical and complex designs, identification of the appropriate level for tests and full reporting of outcomes
☒	☐	Estimates of effect sizes (e.g. Cohen's *d*, Pearson's *r*), indicating how they were calculated

Our web collection on statistics for biologists contains articles on many of the points above.

Software and code

Policy information about availability of computer code

Data collection	SerialEM 3.8
Data analysis	Relion3.1, Coot 0.8.9, Phenix 1.19.2, Pymol 2.4.2, Chimera 1.13, ChimeraX 1.4, CryoSPARC 3.3.0, crYOLO 1.9.6, MotionCor2 1.2.1, Graphpad Prism 8, HOLE 2.3.1, Mascot 2.7.0

For manuscripts utilizing custom algorithms or software that are central to the research but not yet described in published literature, software must be made available to editors and reviewers. We strongly encourage code deposition in a community repository (e.g. GitHub). See the Nature Portfolio guidelines for submitting code & software for further information.

Data

Policy information about availability of data

All manuscripts must include a data availability statement. This statement should provide the following information, where applicable:

- Accession codes, unique identifiers, or web links for publicly available datasets
- A description of any restrictions on data availability
- For clinical datasets or third party data, please ensure that the statement adheres to our policy

The cryo-EM density maps of the human CNGA2/A4/B1b-CaM and CNGA2/A4/B1b-cAMP complex have been deposited in the Electron Microscopy Data Bank under accession numbers EMD-64395 and EMD-64394, respectively. Atomic coordinates have been deposited in the Protein Data Bank under accession numbers 9UPG

for the CNGA2/A4/B1b-CaM structure and 9UPF for the CNGA2/A4/B1b-cAMP structure. The cross-linking mass spectrometry data have been deposited to the ProteomeXchange Consortium via the MassIVE repository with the dataset identifier PXD063119.

Research involving human participants, their data, or biological material

Policy information about studies with human participants or human data. See also policy information about sex, gender (identity/presentation), and sexual orientation and race, ethnicity and racism.

Reporting on sex and gender	N/A
Reporting on race, ethnicity, or other socially relevant groupings	N/A
Population characteristics	N/A
Recruitment	N/A
Ethics oversight	N/A

Note that full information on the approval of the study protocol must also be provided in the manuscript.

Field-specific reporting

Please select the one below that is the best fit for your research. If you are not sure, read the appropriate sections before making your selection.

☒ Life sciences ☐ Behavioural & social sciences ☐ Ecological, evolutionary & environmental sciences

For a reference copy of the document with all sections, see nature.com/documents/nr-reporting-summary-flat.pdf

Life sciences study design

All studies must disclose on these points even when the disclosure is negative.

Sample size	The data size for cryo-EM was determined by the availability of the microscope time and the particle density on the grids. Sufficient cryo-EM datas were collected to achieve the reported resolution of map, which is sufficient for model building.
Data exclusions	CryoEM data processing involved removing poor-quality or damaged particles to achieve high resolution maps through pre-established standard data classification procedures.
Replication	For functional analysis, each experiment was reproduced at least two times on separate occasions. Experimental findings were reliably reproduced.
Randomization	Randomization was not necessary as the independent variables to be tested were sufficient for the functional interpretations within this study. i.e. WT vs mutant current recordings
Blinding	All experiments are not blind. Blinding is not necessary for the purposes of structural determination. For functional analysis, blinding was not necessary due to the quantitative nature of the experiment.

Reporting for specific materials, systems and methods

We require information from authors about some types of materials, experimental systems and methods used in many studies. Here, indicate whether each material, system or method listed is relevant to your study. If you are not sure if a list item applies to your research, read the appropriate section before selecting a response.

Materials & experimental systems

n/a	Involved in the study	
☒	☐	Antibodies
☐	☒	Eukaryotic cell lines
☒	☐	Palaeontology and archaeology
☒	☐	Animals and other organisms
☒	☐	Clinical data
☒	☐	Dual use research of concern
☒	☐	Plants

Methods

n/a	Involved in the study	
☒	☐	ChIP-seq
☒	☐	Flow cytometry
☒	☐	MRI-based neuroimaging

Eukaryotic cell lines

Policy information about cell lines and Sex and Gender in Research

Cell line source(s)	Human embryonic kidney (HEK293 GnTi-) purchased from ATCC and Sf9 insect cells purchased from Thermo Fisher Scientific.
Authentication	No further authentication was performed for commercially available cell lines.
Mycoplasma contamination	Cell lines were not tested for mycoplasma contamination.
Commonly misidentified lines (See ICLAC register)	None.

Plants

Seed stocks	*Report on the source of all seed stocks or other plant material used. If applicable, state the seed stock centre and catalogue number. If plant specimens were collected from the field, describe the collection location, date and sampling procedures.*
Novel plant genotypes	*Describe the methods by which all novel plant genotypes were produced. This includes those generated by transgenic approaches, gene editing, chemical/radiation-based mutagenesis and hybridization. For transgenic lines, describe the transformation method, the number of independent lines analyzed and the generation upon which experiments were performed. For gene-edited lines, describe the editor used, the endogenous sequence targeted for editing, the targeting guide RNA sequence (if applicable) and how the editor was applied.*
Authentication	*Describe any authentication procedures for each seed stock used or novel genotype generated. Describe any experiments used to assess the effect of a mutation and, where applicable, how potential secondary effects (e.g. second site T-DNA insertions, mosiacism, off-target gene editing) were examined.*
